# Effect of Electroacupuncture on Activation of p38MAPK in Spinal Dorsal Horn in Rats with Complete Freund's Adjuvant-Induced Inflammatory Pain

**DOI:** 10.1155/2012/568273

**Published:** 2011-08-08

**Authors:** Yi Liang, Jian-Qiao Fang, Jun-Ying Du, Jun-Fan Fang

**Affiliations:** The Third Clinical Medical College, Zhejiang Chinese Medical University, 548 Binwen Road, Binjiang District, Hangzhou City, Zhejiang Province 310053, China

## Abstract

Activation of mitogen-activated protein kinases (MAPKs), especially p38 MAPK, plays an important role in the development of central sensitization related to persistent inflammatory pain. Electroacupuncture (EA) is well known to relieve persistent inflammatory pain. However, little is known about relationship between EA and p38 MAPK. Inflammatory pain rat model was induced by intraplantar injection of complete Freund's adjuvant (CFA). Male adult SD rats were randomly divided into the saline group, CFA group, and CFA + EA group. EA (constant saquare wave, 2 Hz and 100 Hz alternating frequencies, intensities ranging from 1 to 2 mA) was applied to bilateral “Zusanli” (ST 36) and “Kunlun” acupoints (BL 60) for 30 min, once per day. The paw edema and paw withdrawal threshold (PWT) were measured at preinjection and days postinjection 1, 3, and 14. Spinal p-p38MAPK- immunoreactivty (p-p38MAPK-IR) cells were detected by immunohistochemistry at postinjection day 3 and 14. EA significantly inhibited paw edema at postinjection days 14 and increased PWT at postinjection days 3 and 14. Moreover, the increasing number of spinal p-p38MAPK-IR cells which was induced by CFA injection was suppressed by EA stimulation. These results indicate that anti-inflammatory and analgesic effect of EA might be associated with its inhibition of spinal p38 MAPK activation and thereby provide a potential mechanism for the treatment of inflammatory pain by EA.

## 1. Introduction

The mitogen-activated protein kinases (MAPKs) are important for intracellular signal transduction and play pivotal roles in mediating the generation and maintenance of pain [1]. To date, six distinct groups of MAPKs have been characterized in mammals: extracellular-signal-regulated kinase (ERK), p38, c-jun N-terminal kinase (JNK), ERK7/8, ERK3/4, and ERK5 [2]. Recent data demonstrate that MAPKs, p38 in particular, can be activated in the spinal cord by peripheral and spinal noxious stimuli [3]. It is also shown that phosphorylated p38 (p-p38) MAPK, the active form of p38 MAPK, increases in spinal cord after peripheral inflammation which is induced by CFA [4], bee-venom [5], formalin [3, 6], or capsaicin [7]. Moreover, intrathecal administration of a p38 MAPK inhibitor into spinal cord has been shown to effectively reduce pain behavior associated with the peripheral inflammation [3, 8–10]. 

Electroacupuncture (EA), as a traditional complementary and alternative medicine approach, has been used for several decades in the treatment of many acute and chronic inflammatory diseases [11]. Accumulative evidence demonstrates that EA significantly inhibits paw inflammation and hyperalgesia in a rat model [12–14]. However, it has been reported that EA may produce differential effects under healthy and pathological conditions [15]. The underlying mechanisms of EA analgesia are still not completely understood, and the influence of EA on p38 MAPK activation in spinal cord which is associated with inflammatory pain is unclear. Injection of complete Freund's adjuvant (CFA) into a rat's hind paw provides a very good animal model to study the mechanism of inflammatory pain [16]. Thus, we used a rat CFA model to observe the anti-inflammatory and analgesic effect of EA and to investigate whether EA may relieve inflammatory pain by suppressing the activation of spinal p38 MAPK in CFA-inflamed rats.

## 2. Materials and Methods

### 2.1. Animals

Male Sprague-Dawley rats weighing 180–220 g were provided by the Department of Animal Sciences of our university. They were housed five per cage with food pellets and water ad libitum. Prior to experimental manipulation, rats were allowed to acclimate to the housing facilities for one week and maintained on a 12 : 12 h light-dark cycle and a constant room temperature of 25 ± 2°C. All rats in this study were used strictly in accordance with the National Institutions of Health Guide for the Care and Use of Laboratory Animals in order to minimize the number of animals used and their suffering.

### 2.2. Establishment of Model and Experimental Groups

Inflammatory pain rat model was induced by an injection of 100 *μ*L CFA (Sigma, USA) into the plantar surface of right hind paw. The rats were separated randomly into 3 groups: (1) the saline group with saline injection (same volume as used for the CFA injection); (2) the CFA group with CFA injection, immobilization, and without EA stimulation; (3) the CFA + EA group with CFA injection, immobilization but treated with EA. The rats of the CFA group were also immobilized gently by assistants' hands for 30 min every day, which simulate holder stress in rats of CFA + EA group. Ten rats for each group were included for assessment of inflammation and behavioral test. Three rats were included at each survival time point for immunohistochemical analyses.

### 2.3. EA Stimulation

Rats were loosely immobilized by assistants' hands. Four stainless steel acupuncture needles of 0.25 mm in diameter were inserted a depth of 5 mm into bilateral “Zusanli” (ST36, 5 mm lateral to the anterior tubercule of the tibia) and “Kunlun” (BL60, at the ankle joint level and between the tip of the external malleolus and tendo calcaneus) acupoints. It has been showed that these two acupoints were good at treating inflammatory pain [17]. The two ipsilateral needles were connected with the output terminals of the HANS Acupuncture point Nerve Stimulator (LH-202H, Huawei Co., Ltd., Beijing, China). The EA parameters were set as follows: constant square wave current output (pulse width: 0.6 ms at 2 Hz, 0.2 ms at 100 Hz); intensities ranging from 1 to 2 mA (each intensity for 15 min, totaling 30 min); to 2 Hz and 100 Hz alternating frequencies (automatically shifting between 2 Hz and 100 Hz stimulation for three seconds each). The stimulation was given for 30 min, once per day, and started at day 1 after injection when the assessment of inflammation and behavioral test have been finished.

### 2.4. Assessment of Inflammation

Paw volume (to assess the severity of inflammation) was measured by a water displacement plethysmometer (LYS-7A, Shandong Medical Instrument Factory, China). The hind paw was immersed in a chamber containing electrolyte solution up to the boundary between hairy and nonhairy skin, and the volume displacement was determined electronically. Paw volume was measured in duplicate before CFA/saline injection (as basal paw volume) and 1, 3, and 14 days after CFA/saline injection. Paw edema was calculated as follows: paw edema (ml) = *V*_*t*_ − *V*_0_, where *V*_*t*_ is the paw volume after CFA or saline injection and *V*_0_ is basal paw volume.

### 2.5. Behavioral Test

The paw withdrawal threshold (PWT) was used to assess the inflammatory pain. Rats were placed on a metal mesh table and adapted to the new environment. The mechanical stimulus was delivered to the plantar surface of right hind paw from below the floor of the test chamber by an automated testing device (dynamic plantar aesthesiometer, Ugo Basile, Italy). A steel rod (diameter of 0.5 mm) was pushed against the hind paw with ascending force. The force went from 0 to 50 g over a 20 s period. When the animal withdrew its hind paw, the mechanical stimulus was automatically stopped, and the force at which the animal withdrew its paw was recorded as PWT. Withdrawal responses were taken from four consecutive trials with at least 1min between trials and averaged.

### 2.6. Immunohistochemistry

Animals were deeply anesthetized with 10% choral hydrate (0.35 mL/100 g, ip) and transcardially perfused with 150 mL cold sterilized saline followed by 500 mL cold, fresh 4% paraformaldehyde in 0.1 M phosphate-buffered saline (PBS). The L_4_–L_6_ segments were removed and postfixed in the same fixative for 6 h at 4°C before transfer to 15–30% sucrose for cytoprotection. Transverse 30 *μ*m thick sections were cut on a cryostat and stored as free-floating sections in 0.1 M PBS (containing 30% sucrose, 30% ethylene glycol) at −20°C. Immunochemistry was performed on free-floating spinal cord section. Endogenous peroxidase activity was quenched with 15 min incubation in 3% H_2_O_2_ at 37°C. Sections were blocked in 5% normal goat serum for 30 min at room temperature and then transferred to primary antibody solution containing rabbit anti rat p-p38MAPK (1 : 200, CST, USA) for overnight at 4°C. Sections were washed and placed in biotinylated secondary antibody (goat antirabbit, 1 : 400) for 1 h at 37°C. Following incubation with HRP-conjugated avidin (1 : 400) for 1 h at 37°C, sections were incubated with DAB substrate for 40 seconds. Sections were mounted on glass slides, dehydrated through an ascending series of alcohols, cleared with xylene, and coverslipped. Images were captured from ipsilateral dorsal horn at 20 × magnification using a Leica CCD camera. In a given area (200 *μ*m × 150 *μ*m) that is located in superficial spinal cord (laminae I-II), the number of p-p38 MAPK positive cells was counted automatically using Image Pro Plus 6.0 under blinded conditions. A minimum of five tissue sections/animal with a minimum of three animals/group were counted.

### 2.7. Statistical Analysis

Data are expressed as mean ± standard deviation (SD). The differences among groups were assessed by one-way analysis of variance (ANOVA) and followed by LSD post hoc test for the normal distribution and homogeneity of variance data. *P* < 0.05 was considered statistically significant.

## 3. Results

### 3.1. Effect of EA on Ipsilateral Paw Edema in CFA Rats

**Figure 1:**
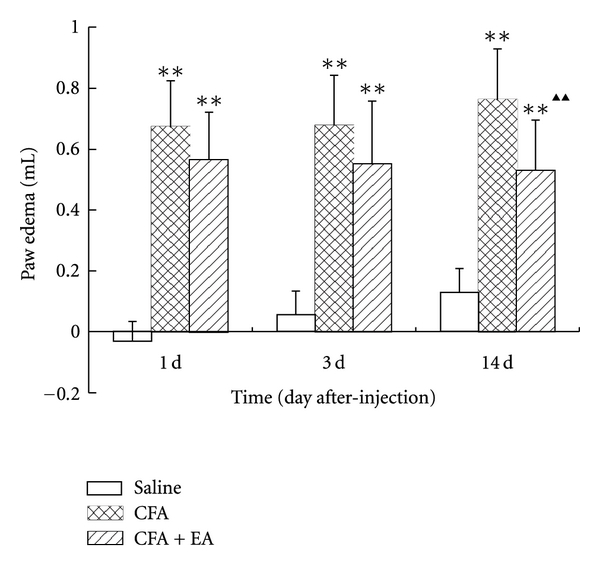
Effect of electroacupuncture (EA) on ipsilateral paw edema at different time points in complete Freund's adjuvant (CFA) rats. Values are mean ± SD, *n* = 10 animals per experimental group. **P < 0.01 versus saline group; ^▲▲^ *P* < 0.01 versus CFA group at corresponding time points.

Basal paw volumes in the saline, CFA, and CFA + EA group were 1.455 (±0.129), 1.448 (±0.161), 1.555 (±0.089) mL, respectively (*P* > 0.05). As shown in Figure 1, the paw edema in CFA group and CFA + EA group was higher than that in saline group at all time points (*P* < 0.01). The paw edema in CFA group increased gradually whereas that in CFA + EA group decreased slightly. Compared with the CFA group at corresponding time points, the paw edema in the CFA + EA group markedly decreased at postinjection day 14 (*P* < 0.01).

### 3.2. Effect of EA on Ispilateral PWTs in CFA Rats

**Figure 2:**
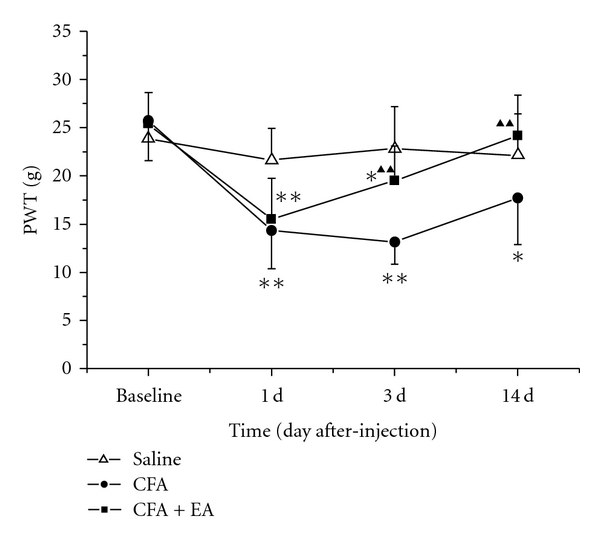
Effect of EA on ipsilateral paw withdrawal thresholds (PWTs) at different time points in CFA rats. Values are mean ± SD, *n* = 10 animals per experimental group. *P < 0.05, **P < 0.01 versus saline group; ^▲▲^ *P* < 0.01 versus CFA group at corresponding time points.

As shown in Figure 2, no difference in basal PWTs among groups was observed before saline or CFA injection. However, at day 1, 3, and 14 after CFA injection, the PWTs in the CFA group were obviously lower than those in the saline group (*P* < 0.01 or *P* < 0.05). After EA stimulation, the rats' PWTs were increased significantly as compared with the CFA group at postinjection days 3 and 14 (*P* < 0.01).

### 3.3. Effect of EA on Phosphor-p38MAPK-Immunoreactivity (p-p38MAPK-IR) Expression in the Ipsilateral Spinal Dorsal Horn in CFA Rats

**Figure 3:**
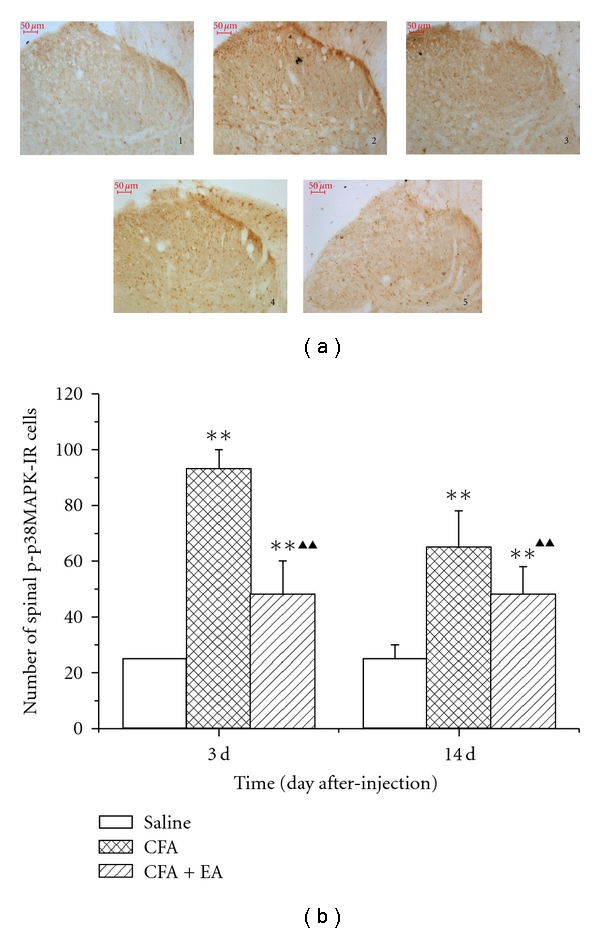
Effect of EA on phosphor-p38MAPK-immunoreactivty (p-p38MAPK-IR) expression in the ipsilateral spinal dorsal horn induced in CFA rats. (a) Representative sections of p-p38MAPK-IR cells in the ipsilateral spinal dorsal horn at postinjection days 3 and 14. (section  1, saline group; sections  2 and 4, CFA group, sections  3 and 5, CFA + EA group; sections  2 and 3, postinjection day 3; sections  4 and 5, postinjection day 14; Bars = 50 *μ*m; section thickness = 30 *μ*m). (b) Quantification of p-p38MAPK-IR showing that EA suppressed p-p38MAPK-IR expression in the ipsilateral spinal dorsal horn. Values are mean ± SD, *n* = 3 animals per experimental group. **P < 0.01 versus saline group; ^▲▲^ *P* < 0.01 versus CFA group at corresponding time points.

CFA injection induced an increase in number and intensity of p-p38MAPK-IR cells in the spinal dorsal horn ipsilateral to inflammation. However, a low basal constitutive expression of p-p38MAPK-IR was also observed in the saline group (Figure 3(a)). The number of p-p38MAPK-IR cells was detected increasingly at day 3 after CFA injection, and the significant level was maintained until 14 days (*P* < 0.01). After EA stimulation, the number of p-p38MAPK-IR cells in ipsilateral spinal dorsal horn was decreased markedly as compared with the CFA group at postinjection days 3 and 14 (*P* < 0.01) (Figure 3(b)).

## 4. Discussion

The present study demonstrates, for the first time, that the anti-inflammatory and analgesic effects of EA were associated with the activation of p38MAPK in spinal dorsal horn. In the CFA-induced inflammatory pain model, the paw edema and the number of p-p38MAPK-IR cells in ipsilateral spinal dorsal horn were significantly increased, while the pain threshold was decreased. Treatment with EA obviously ameliorated CFA-induced paw edema and hyperalgesia and decreased the number of p-p38MAPK-IR cells in spinal dorsal horn, indicating that the p38MAPK signaling pathway might play an important part in the anti-inflammatory and analgesic effect of EA.

Recent reports have demonstrated that activation of p38 MAPK within the spinal cord has been implicated in a variety of enhanced pain states [8, 18–24] and plays a pivotal role in creation of persistent pain. Increasing activation of p38 MAPK in spinal cord has been observed not only at peripheral nerve injury caused by chronic constriction injury (CCI) [8, 23] and spinal nerve ligation (SNL) [18–20], but also at peripheral inflammation induced by CFA [4], bee-venom [5], formalin [3, 6], or capsaicin [7]. It has been reported that p-p38 MAPK levels in lumbar enlargements increased in individual rats between days 8 and 17 after CFA immunization by western blot analysis. Moreover, it is shown that numerous cells stained positively for p-p38 MAPK in the dorsal horn of CFA-immunized rats, whereas the control rats contained only a few scattered p-p38MAPK-positive cells [4]. There was a rapid increase in phosphorylated p38 MAPK in spinal cord following intrathecal administration of substance P or intradermal injection of formalin. Immunocytochemistry also revealed that phosphorylated-p38-MAPK-immunoreactive cells were predominantly present in laminae I–IV of the dorsal horn [3]. Consistent with these previous studies in the inflammatory pain model, we have shown that activation of p38 MAPK in ipsilateral spinal dorsal horn was detected on day 3 after CFA injection and was maintained for two weeks.

Accumulating evidence has shown that activation of p38 MAPK in the dorsal horn, which contributes to the pathogenesis of pain, was expressed exclusively in microglia, but not in neurons or astrocytes. Gu et al. have shown a marked increase of the level of p-p38 MAPK in the microglia of dorsal horn in CCI model [23]. Moreover, similar results have also been found in the SNL model [18–20]. Svensson et al. have reported that activated p38 MAPK is located in spinal microglia, but not in neurons or other glia after intrathecal administration of SP [3]. In contrast to the present findings with peripheral nerve injury and central inflammatory stimulation, Hua et al. have shown that after carrageenan paw injection, the increased p-p38 immunoreactivity was seen primarily in microglia but also in a small population of neurons [25]. Taking these results together, we speculate that it remains to be investigated how p38 MAPK activation in spinal microglia contributes to CFA-induced pain sensitization.

Acupuncture therapy, especially EA, has been accepted worldwide mainly for the treatment of acute and chronic pain [26–29]. In the present study, we observed that EA has suppressed paw edema induced by intraplantar CFA. This is supported by previous studies reporting that EA significantly inhibits edema compared to sham EA control in CFA-injected rat [12, 14, 30]. A previous study reported that a single or repetitive EA could reduce mechanical hyperalgesia, but not thermal hyperalgesia, in CFA-inflammatory pain rats [31]. Consistent with this, we also found that EA increased paw withdrawal threshold in CFA rats at postinjection days 3 and 14. Although great progress has been made in recent years in investigating analgesic mechanisms of acupuncture, the influence of acupuncture on signal molecules and signal pathways remains elusive. A recent study has reported that pre-EA significantly decreased p-p38 MAPK protein expression in the spinal dorsal horn of rats suffering from visceral pain [32]. Our present study demonstrated for the first time that repeated EA significantly suppressed CFA-induced activation of p38MAPK in spinal dorsal horn at postinjection days 3 and 14. Further investigation is required to determine the exact effect of the MAPK signal pathway on the spinal cord of rats suffering from the peripheral inflammatory pain.

In conclusion, EA has anti-inflammatory and analgesic effect on CFA-induced inflammatory pain, which might be associated with its inhibition of spinal p38 MAPK activation. Spinal p38 MAPK, exactly p38 MAPK in microglia, and other members of the p38 MAPK signal transduction pathway represent promising targets for the treatment of inflammatory pain by EA.
